# Norma Latina Neuropsychological Evaluation in Individuals with Multiple Sclerosis and Its Relationship with Disability

**DOI:** 10.3390/brainsci15121251

**Published:** 2025-11-21

**Authors:** Adriana Aguayo-Arelis, Brenda Viridiana Rabago-Barajas, Alina Mariela Cárdenas Gómez, Jesús Emmanuel Arana Yepez, Ana Miriam Saldaña-Cruz, Alberto Fragoso-Ruiz

**Affiliations:** 1 Department of Applied Psychology, University Center for Health Sciences, University of Guadalajara, Guadalajara 45100, Jalisco, Mexico; adriana.aguayo@academicos.udg.mx (A.A.-A.); alina.cardenas5227@academicos.udg.mx (A.M.C.G.); 2 Pharmacology and Behavior Laboratory, Neuroscience Institute, University Center of Biological and Agricultural Sciences, University of Guadalajara, Guadalajara 45100, Jalisco, Mexico; yepezneal@gmail.com; 3 Institute of Experimental and Clinical Therapeutics, Department of Physiology, University Center for Health Sciences, University of Guadalajara, Guadalajara 45100, Jalisco, Mexico; ana.saldanac@academicos.udg.mx; 4 Master’s Program in Educational Psychology, Department of Applied Psychology, University Center for Health Sciences, University of Guadalajara, Guadalajara 45100, Jalisco, Mexico; alberto.fragoso3867@alumnos.udg.mx

**Keywords:** neuropsychological evaluation, cognitive dysfunction, multiple sclerosis, disability

## Abstract

**Background:** Multiple Sclerosis (MS) is an inflammatory, autoimmune and neurodegenerative disease of the central nervous system that leads to the progressive loss of motor and sensory functions. Cognitive dysfunction is a common symptom that significantly affects quality of life and daily activities. The MS diagnosis involves progressive disability due to its neurodegenerative nature. **Objective:** to analyze the relationship between the Latin Norm Neuropsychological Assessment and Disability in Multiple Sclerosis (NLNAMS) battery and physical disability in patients with MS. **Methods:** A retrospective review of 100 medical records was conducted. Three sections of clinical information were analyzed: (1) sociodemographic data and medical history, (2) neurological examination including disability measures using the Expanded Disability Status Scale (EDSS) and the Multiple Sclerosis Severity Score (MSS), and (3) neuropsychological assessment results obtained through the NLNAMS battery to evaluate cognitive functioning across multiple domains. **Results:** High correlations were observed between EDSS scores and performance on the Symbol Digit Modalities Test (SDMT) and the Hopkins Verbal Learning Test–Revised (HVLT-R), which assess attention, processing speed and memory. Strong correlations were also found between EDSS and performance on verbal fluency tests, Trail Making Test (TMT), Rey–Osterrieth Complex Figure copy (ROCF), and the Modified Wisconsin Card Sorting Test (M-WCST). No significant correlation with MSS was found. **Conclusions**: The neuropsychological evaluation conducted with the NLNAMS battery showed a relationship between physical disability in multiple sclerosis and the domains of attention, processing speed, and memory. Therefore, this battery may provide valuable information for disease monitoring and prognosis.

## 1. Introduction

Multiple Sclerosis (MS) is an inflammatory, autoimmune, and neurodegenerative disease of the central nervous system that leads to progressive loss of motor and sensory functions. Beyond its physical manifestations, cognitive deterioration is one of the most frequent and disabling symptoms, significantly affecting patients’ quality of life and daily functioning [1]. The neurodegenerative nature of MS results in cumulative neurological damage and a wide spectrum of symptoms, including motor, sensory, visual, and balance disturbances, as well as mood and cognitive disorders [2,3]. MS is, after traumatic brain injury, the leading cause of disability among young adults. This disability emerges early in the disease course due to widespread demyelination and axonal injury in the central nervous system. The topographical model of MS describes this progressive accumulation of neurological damage and its contribution to increasing levels of disability over time [4].

The Expanded Disability Status Scale (EDSS) is the most widely used measure of disability in MS. It ranges from 0 (normal neurological function) to 10 (death due to MS) in 0.5-unit increments and primarily evaluates motor and sensory domains, with higher scores mainly determined by walking impairment. Although useful for monitoring disease progression, the EDSS has been criticized for underrepresenting non-motor aspects of disability, particularly cognitive dysfunction [5].

To complement this limitation, the Multiple Sclerosis Severity Score (MSSS) was developed as a derivative measure that adjusts the EDSS by disease duration, thereby estimating the relative rate of disability progression for each patient [6]. Lower MSSS values (≤0.2) are generally associated with benign courses, while higher scores (≥1.4) reflect more aggressive disease trajectories. Despite their conceptual relationship, EDSS and MSSS are not always strongly correlated, as they capture distinct aspects of disease burden: EDSS represents the current physical disability status, whereas MSSS reflects disability severity relative to disease duration. Evaluating both scales may therefore provide a more comprehensive understanding of the relationship between cognitive impairment and physical disability in MS.

Cognitive impairment is one of the most prevalent and disabling manifestations of MS, affecting approximately 60–70% of patients [7,8]. These deficits primarily result from demyelination and diffuse axonal damage in both white and gray matter, leading to altered efficiency in neural networks that support memory, attention, executive functioning, and information processing. Despite its clinical relevance, cognitive dysfunction remains frequently underrecognized in standard neurological evaluations, largely because traditional disability scales, such as the EDSS, focus predominantly on motor and sensory domains. This underscores the need for neuropsychological instruments capable of capturing the multidimensional impact of MS on cognition, particularly in culturally diverse populations.

Although several cognitive assessment tools are available to evaluate cognition in MS, there is still variability in their clinical use across countries, despite the existence of international and national guidelines for cognitive assessment in MS. In Latin America, however, there remains limited information on instruments that have been validated and standardized for local populations.

To address this gap, our group previously developed and standardized the NLNAMS, a battery comprising ten neuropsychological tests that provide culturally appropriate normative data for Latin American populations [9]. The initial standardization was conducted in healthy participants from several Latin American countries, including Mexico. Subsequently, the clinical usefulness of the NLNAMS battery in detecting cognitive impairment was evaluated exclusively in Mexican patients with MS.

Given the clinical relevance of cognitive impairment and its potential contribution to overall disease burden, a better understanding of its relationship with disability progression is essential. Therefore, the present study aimed to analyze the association between cognitive performance, as assessed by the NLNAMS, and disability severity, as measured by the EDSS.

## 2. Materials and Methods

**Participants**

We conducted a retrospective analysis of 100 medical records of Mexican patients diagnosed with MS, previously included in the study by Rivera and colleagues (2022). The inclusion/exclusion criteria for were: expedient legible, diagnosis of MS according to McDonald criteria, have signed the informed consent, ≥18 years old, no history of neurological or psychiatric diagnosis (e.g., schizophrenia, schizophreniform, schizoaffective, or delusional disorders), no history of alcohol/drugs abuse, no severe visual and/or hearing deficit, not have had a relapse occurring within the last 30 days, and no treatment with corticosteroids within the last 30 days.

**Procedure and instruments**

A review of 150 medical records of subjects diagnosed with MS was carried out, who were treated at a reference center for MS in Guadalajara, from the beginning of 2018 to 2020. We used a total of 100 medical records which were selected according to the inclusion and exclusion criteria indicated in the participant’s section. For the review of the medical records, we used three sections: Clinical history to collect sociodemographic data and medical history, Neurological examination for the clinical variables as well as disability scores of the EDSS and MS severity score and Neuropsychological test provided by the NLNAMS to obtain cognitive function of each patient.

**Figure 1:**
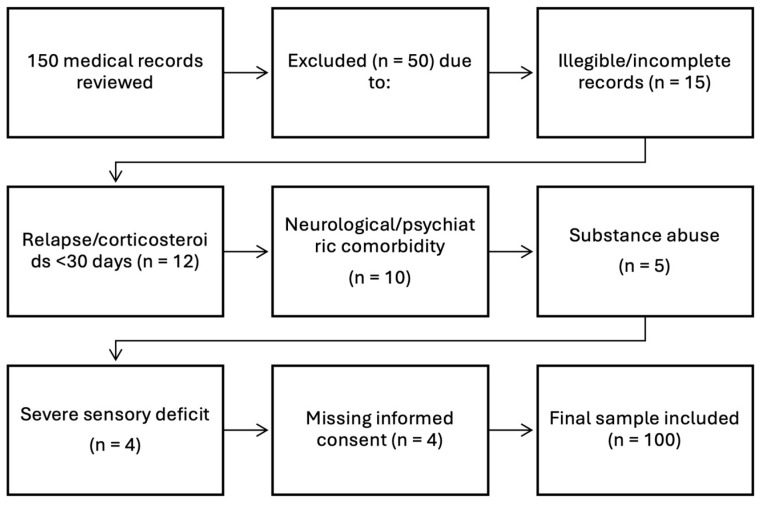
Flowchart of the study sample selection process.

A total of 150 medical records of patients diagnosed with multiple sclerosis were reviewed. Fifty cases were excluded based on the following criteria: illegible or incomplete records (*n* = 15), relapse or corticosteroid treatment within the previous 30 days (*n* = 12), neurological or psychiatric comorbidity (*n* = 10), substance abuse (*n* = 5), severe visual or hearing deficit (*n* = 4), and missing informed consent (*n* = 4). The final sample comprised 100 patients who met all inclusion criteria (Figure 1).

**Neuropsychological test**

Rey–Osterrieth Complex Figure (ROCF) to evaluate visual perception, visual-spatial construction ability and visual memory [10]. Stroop Color and Word Test (Stroop test) to attentional function and the ability to resist verbal interference and level of selective attention [11]. Modified Wisconsin Card Sorting Test (M-WCST) to executive function and cognitive flexibility [12], Trail Making Test (TMT) [13] and Symbol Digit Modalities Test (SDMT) to evaluate attention, executive function, and information processing speed [14], Brief Test of Attention (BTA) to divided auditory attention [15]. Verbal Fluency Test (VFT; in this study, animals, fruits, professions, and letters F/A/S/M were used) [16], Boston Naming Test (BNT) to lexical access ability [17]. Hopkins Verbal Learning Test–Revised (HVLT-R) to short- and long-term verbal memory as well as recall [18].

**Ethics Considerations**

The protocol was approved by the local research ethics committee of centro universitario de ciencias de la salud, of the University of Guadalajara (approval number: CI-05121).

**Data analysis**

Mean and standard deviation were used for quantitative variables, while frequencies and percentages were calculated for qualitative measures. The Shapiro–Wilk test was applied to assess data normality. Pearson’s correlation coefficient was used to examine the relationship between neuropsychological test scores, EDSS, and the MS Severity Score. Analyses were performed using SPSS version 21.

Correlations were analyzed between EDSS and each of the NLNAMS test results. The closer the correlation coefficient is to −1 or +1, the stronger the association between the two variables. The strength of the correlations in this study was interpreted as follows: *r* = 0.10–0.29 = weak, *r* = 0.30–0.39 = moderate, *r* = 0.40–0.49 = strong, and *r* ≥ 0.50 = high [19].

## 3. Results

The sociodemographic data and clinical variables of the disease are shown in Table 1.

Table 2 shows weak correlations between neuropsychological tests such as Stroop word test (information processing speed, ability and speed reading), M-WCST perseverative error phase (behavioral inhibition), ROCF memory test (visual memory) and score EDSS as a measure of disability.

Moreover, Table 3 shows the moderate correlations between disabilities measured with EDSS and neuropsychological tests such as, Stroop test word-color and M-WCST (cognitive flexibility and behavioral inhibition), HVLT-R Recognition (cue use memory), BTA total numbers, total letters, and total numbers and letters (selective auditory attention), HVLT-R Test 1 and 2 (memory verbal), Stroop test colors reading, FV letter F thinking speed, BNT language and TMT-A (attention and information processing speed).

Table 4 shows strong correlations between the EDSS and neuropsychological tests such as, M-WCST Test-total categories correct (executive function), Phonological, verbal and semantic fluency (categorization and language), ROCF-copy (visual memory) and HVLT-R-total (verbal memory).

In addition, Table 5 presents a high correlation between EDSS and neuropsychological tests such as SDMT (attention, concentration and processing information speed) and HVLT-R-Test 3 (verbal memory and learning).

Finally, Table 6 presents no significant differences (no correlation) between the scale to measure disability is the Multiple Sclerosis Severity Score and the Norma Latina neuropsychological assessment.

## 4. Discussion

The present study aimed to analyze the association between cognitive performance and physical disability in Mexican patients with MS, using the NLNAMS battery. This battery, composed of ten neuropsychological tests, was previously standardized in a Mexican population [9] and allows for the evaluation of key cognitive domains commonly affected in MS. Specifically, we examined whether NLNAMS scores were associated with disability severity, as measured by the EDSS, and with the MSSS, which adjusts disability according to disease duration. These complementary indices provide a broader understanding of how cognitive and physical aspects of MS interact within this clinical sample.

Previous studies have examined the relationship between EDSS and cognitive function in MS, with inconsistent results. Caneda and Vecino reported moderate correlations between EDSS and the Brief International Cognitive Assessment for Multiple Sclerosis (BICAMS) [20]. Specifically, EDSS was moderately correlated with the SDMT (*r* = 0.55), which assesses attention and processing speed; the BVMT (*r* = 0.54), which measures visuospatial memory; and the CVLT (*r* = 0.40), which evaluates verbal learning and language. These authors concluded that BICAMS may be suitable for screening and monitoring cognitive impairment in MS. In contrast, other studies have found weaker correlations between EDSS and measures such as the SDMT, BVMT, MoCA, and CVLT, highlighting that despite up to 50% of MS patients presenting with some degree of cognitive deficit, it remains unclear which cognitive domains are most affected [21].

In our study, we observed significant but weak correlations between EDSS and several cognitive domains, including processing speed, inhibitory control, and visuospatial memory. The Stroop test, which primarily measures processing speed and selective attention, showed a weak association with EDSS scores, indicating that slower information processing may accompany greater physical disability. This finding is consistent with previous reports showing that patients with higher EDSS scores tend to perform worse on tests assessing processing speed, such as the SDMT from the BRB-N battery [22]. Similarly, studies using the Paced Auditory Serial Addition Test (PASAT-3) and SDMT have described weak to moderate correlations with markers of brain atrophy [23]. In line with these observations, we also found a weak negative correlation between EDSS and SDMT performance (*r* = −0.58), comparable to that reported by Sadigh-Eteghad [24] who found similar weak correlations between EDSS and SDMT (*r* = −0.496) and PASAT (*r* = −0.248). Together, these findings suggest that slower processing speed often co-occurs with increased physical disability in MS, although the relationship remains correlational rather than causal [24].

We also evaluated visual memory and visuospatial organization using the ROCF, which additionally involves visuoconstructive abilities. These cognitive functions are commonly affected in MS due to demyelinating lesions in temporo-parieto-occipital white matter pathways; however, previous studies have reported alterations in only about 20–26% of patients [25]. In our sample, ROCF performance showed a moderate negative correlation with EDSS (*r* = −0.56), indicating that poorer visuospatial construction and recall were associated with greater disability. One possible explanation for this moderate association is that the ROCF and similar tests may have limited sensitivity to subtle visuospatial or memory deficits. Supporting evidence from the BVMT-R shows that spatial memory is often impaired in MS and correlates moderately with EDSS (*r* = −0.34) [26,27]. Conversely, other studies, such as those employing the SPART 10/36 spatial recall test, have not found significant correlations between spatial learning and EDSS [22]. It is important to consider that performance on visuoconstructive tasks may also be influenced by motor impairment or hand dexterity limitations, which could partly account for the shared variance between cognitive and physical disability measures. Overall, these findings suggest that visuospatial and visuoconstructive dysfunctions are relatively frequent but heterogeneous in MS, reflecting both cognitive and motor components of disease progression.

Regarding the low correlation results, we examined aspects of executive functioning related to inhibitory control using the Stroop and M-WCST tests. Inhibitory control refers to the ability to suppress automatic but inappropriate responses and to filter out irrelevant information when solving a task. In our study, EDSS showed a weak correlation with Stroop performance, suggesting that greater physical disability was modestly associated with reduced inhibitory efficiency. In contrast, performance on the M-WCST, which primarily measures cognitive flexibility and set-shifting, did not show a significant association with EDSS. Similarly, no significant differences in performance were reported between MS patients and controls for ocular motor tasks or PASAT, and these measures were not correlated with EDSS scores [28]. Other studies have also described weak correlations between inhibition measures, such as the Delis–Kaplan Executive Function System (D-KEFS) color–word interference test, and EDSS (*r* = −0.28 to −0.36) [24]. Taken together, these findings suggest that deficits in inhibitory control and related executive processes may occur independently of the degree of physical disability, reinforcing the heterogeneity of cognitive impairment in MS.

We also observed moderate correlations in tests assessing executive function, verbal memory, attention, and language domains frequently associated with frontal lobe activity. The frontal lobes play a key role in cognitive flexibility, hypothesis generation, and abstract reasoning, which are often affected in MS [29,30]. In our study, performance on the M-WCST, Stroop, verbal fluency (phonological and semantic), and TMT-B showed moderate correlations with EDSS scores. These results suggest that greater physical disability tends to coincide with reduced efficiency in executive and attentional control, reflecting the involvement of frontal networks in disease progression. This pattern is consistent with previous studies reporting associations between higher EDSS scores and poorer executive performance [31]. However, given the cross-sectional design, these associations should be interpreted as concurrent relationships rather than predictive effects.

Although frontal lobe-related functions showed moderate correlations, verbal memory and perceptual organization emerged as the domains most strongly associated with EDSS in our sample. The ROCF, which includes multiple stages of visual construction and recall, was particularly informative. The copy phase primarily engages visuospatial and constructional abilities, which tend to decline with disease progression [32]. Visuospatial decline has also been linked to reduced sensorimotor adaptation and slower motor learning, effects that can parallel normal aging processes [33]. Moreover, age has been identified as a relevant factor influencing ROCF performance, even in the copying condition. Patients with neurocognitive disorders generally perform below normative expectations for each ROCF component [34]. Considering that the average age of our participants corresponds to young adulthood, the finding of lower perceptual organization performance may reflect an early compromise of visual–motor integration rather than age-related decline.

Verbal memory is one of the cognitive domains most frequently affected in MS and often shows impairment early in the disease course, even among younger patients with mild cognitive deficits [35]. In the present study, we assessed verbal learning and memory using the HVLT-R. The total recall score demonstrated a moderate correlation with EDSS, approaching the threshold for a strong association. This finding supports previous evidence that verbal learning efficiency may be sensitive to disease-related cognitive changes. Although our data are cross-sectional, the results highlight the relevance of including standardized verbal memory measures in the neuropsychological evaluation of MS patients to better capture subtle cognitive dysfunction.

Finally, attention is particularly susceptible to progressive axonal damage in MS [36]. The TMT-A, BTA, and SDMT are well-established neuropsychological measures frequently used to assess attention and information processing efficiency in MS [37]. In our study, performance on these tests showed moderate correlations with EDSS scores, suggesting that attentional efficiency decreases alongside greater physical disability. This finding is consistent with previous research linking early axonal loss to both neurological and cognitive deterioration [38,39]. These results support the inclusion of attentional measures in clinical follow-up protocols, as they may contribute to a more comprehensive characterization of disease impact.

### Limitations

This cross-sectional study does not allow assessment of the longitudinal progression of cognitive dysfunction and disability in patients with multiple sclerosis. Additionally, the patient sample corresponds only to a population from the western region of the country. Although clinical phenotype and treatment data were collected, these variables were not included in the present analysis because they were not significant in our previous study [9] and the small number of cases in some subgroups (e.g., progressive forms or less frequent treatments) did not allow reliable statistical comparisons. Future research should incorporate these factors to explore potential interactions with cognitive performance and disability. Finally, neuroimaging data were not available to corroborate the findings with neurophysiological markers.

Moreover, as multiple correlations were explored, no correction for multiple comparisons was applied. Therefore, the results should be interpreted with caution and considered exploratory, requiring confirmation in future studies.

Nevertheless, this study provides valuable evidence using a culturally adapted neuropsychological battery (NLNAMS) to assess cognitive function in Mexican patients with MS, allowing examination of its association with physical disability as measured by the EDSS.

## 5. Conclusions

In conclusion, our findings demonstrate significant relationships between cognitive performance particularly in the domains of processing speed, attention, and memory and physical disability, as measured by the EDSS, in Mexican patients with multiple sclerosis. These results are consistent with previous research and extend current knowledge by employing a culturally validated neuropsychological battery (NLNAMS) specifically developed for Latin American populations.

Although the correlations observed were mostly moderate, they underscore the importance of incorporating cognitive assessments into routine clinical evaluations, as disability measures based solely on motor or sensory function may underestimate the overall impact of the disease. Given the heterogeneity of multiple sclerosis and the variability in cognitive impairment across studies, future research using larger and longitudinal samples is warranted to clarify whether cognitive measures can serve as prognostic indicators of disability progression.

Overall, our study supports the inclusion of standardized neuropsychological evaluations in clinical practice to improve the characterization of multiple sclerosis and enhance patient care.

## Figures and Tables

**Table 1:** Patient demographic and clinical data.

Patients Characteristics	
Number of patients	100
Sex	
Female	69%
Male	31%
Years of education	13.7 +/− 4.26
Age	39 +/− 11.1
Time elapsed from first symptom to diagnosis	2.13 +/− 2.87
Years of disease evolution	7.68 +/− 6.62
Relapses during the last year	0.57 +/− 0.79
EDSS	3.68 +/− 1.69
MSSS	1.07 +/− 1.26
Clinical subtype	Relapsing–remitting: 93 Secondary progressive: 5 Primary progressive: 2
Disease-modifying treatment	Glatiramer acetate: 83 Interferon: 12 Azathioprine: 3 No treatment: 2

Note: EDSS = Expanded Disability Status Scale; MSSS = Multiple Sclerosis Severity Score. Data are presented as mean ± standard deviation or percentage (%).

**Table 2:** Weak correlation between Norma Latina neuropsychological assessment and Expanded Disability Status Scale.

Test	*p*-Value	r-Value
Stroop Test. Words	0.016	−0.262 *
M-WCST Perseverative Error	0.005	0.289 **
ROCF memory	0.005	−0.292 **

Note: STROOP TEST = Stroop Color and Word Test; M-WCST = Modified Wisconsin Card Sorting Test; ROCF = Rey-Osterrieth Complex Figure. * *p* < 0.05, ** *p* < 0.01.

**Table 3:** Moderate correlation between Norma Latina neuropsychological assessment and Expanded Disability Status Scale.

Test	*p*-Value	r-Value
M-WCST. Total error	0.003	0.307 **
Stroop Test. Word-color	0.003	−0.323 **
HVLT-R. Recognition	<0.001	−0.327 ***
BTA. Numbers total	<0.001	−0.340 ***
HVLT-R assay 1	<0.001	−0.341 ***
HVLT-R assay 2	<0.001	−0.357 ***
BTA. Words total	<0.001	−0.359 ***
Stroop Test. Colors	<0.001	−0.362 **
FV. F Word	<0.001	−0.362 ***
TBA. Words and numbers total	<0.001	−0.383 ***
BNT	<0.001	−0.386 ***
TMT-A	<0.001	0.393 ***

Note: M-WCST = Modified Wisconsin Card Sorting Test; STROOP TEST = Stroop Color and Word Test; HVLT-R = Hopkins Verbal Learning Test–Revised; BTA = Brief Test of Attention; FV = Verbal Fluency Test; BNT = Boston Naming Test; TMT = Trail Making Test. ** *p* < 0.01, *** *p* < 0.001.

**Table 4:** Strong correlation between Norma Latina neuropsychological assessment and Expanded Disability Status Scale.

Test	*p*-Value	r-Value
M-WCST. Total categories corrects	<0.001	−0.402 ***
FV-Fruits	<0.001	−0.406 ***
FV-Word M	<0.001	−0.408 ***
FV-Animals	<0.001	−0.411 ***
FV-Jobs	<0.001	−0.419 ***
FV-Word S	<0.001	−0.440 ***
FV-Word A	<0.001	−0.447 ***
TMT-B	<0.001	0.449 ***
ROCF-copy	<0.001	0.458 ***
HVLT-R Total	<0.001	−0.466 ***

Note: M-WCST = Modified Wisconsin Card Sorting Test; FV = Verbal Fluency Test; TMT = Trail Making Test; ROCF = Rey–Osterrieth Complex Figure; HVLT-R = Hopkins Verbal Learning. *** *p* < 0.001.

**Table 5:** High correlation between Norma Latina neuropsychological assessment and Expanded Disability Status Scale.

Test	*p*-Value	r-Value
SDMT	<0.001	−0.549 ***
HVLT-R Assay 3	<0.001	−0.573 ***

Note: SDMT = Symbol Digit Modalities Test; HVLT-R = Hopkins Verbal Learning. *** *p* < 0.001.

**Table 6:** Correlation Norma Latina neuropsychological assessment and scale to measure disability is the Multiple Sclerosis Severity Score.

Test	*p*-Value	r-Value
HVLT-R assay 1	0.580	0.056
HVLT-R assay 2	0.380	−0.089
HVLT-R assay 3	0.335	−0.098
HVLT-R Total	0.748	−0.033
HVLT-R Recognition	0.475	0.073
ROCF Copy	0.224	−0.128
ROCF Memory	0.365	−0.096
BTA Number total	0.339	−0.097
BTA Word total	0.060	−0.190
TBA TOTAL	0.116	0.159
SDMT	0.739	−0.034
TMT-A	0.113	0.166
TMT-B	0.180	0.140
STROOP TEST words	0.733	−0.038
STROOP TEST colors	0.609	−0.057
STROOP TEST PC	0.820	0.025
STROOP TEST interference	0.346	0.104
FV word F	0.727	−0.036
FV word A	0.119	−0.158
FV word S	0.081	−0.176
FV word M	0.180	−0.136
FV Animals	0.998	−0.000
FV Fruits	0.963	−0.005
FV Jobs	0.119	−0.158
M-WCST complete categories	0.102	−0.171
M-WCST Perseverative Errors	0.361	−0.096
M-WCST Total Errors	0.188	0.139
M-WCST error percentage	0.972	0.004
BNT-long	0.375	0.019

Note: HVLT-R = Hopkins Verbal Learning; ROCF = Rey–Osterrieth Complex Figure; BTA = Brief Test of Attention; SDMT = Symbol Digit Modalities Test; TMT = Trail Making Test; STROOP TEST = Stroop Color and Word Test; FV = Verbal Fluency Test; M-WCST = Modified Wisconsin Card Sorting Test; BNT = Boston Naming Test.

## Data Availability

The data presented in this study are available on reasonable request from the corresponding author. The data are not publicly available due to privacy and ethical restrictions related to patient confidentiality.
